# Palliative Care for Childhood Cancer

**DOI:** 10.3390/children9060777

**Published:** 2022-05-25

**Authors:** Erna M. Michiels

**Affiliations:** Princess Máxima Center for Pediatric Oncology, 3584 CS Utrecht, The Netherlands; e.michiels@prinsesmaximacentrum.nl

Cure rates for children with cancer are improving, but often at the cost of quality of life during treatment. Additionally, although cure rates are improving, 20–25% of children still die from the disease or complications. 

Palliative care in pediatric oncology is perceived as a difficult combination. Pediatric palliative care specialists want to be involved sooner in the disease trajectory to be able to focus on quality of life more and earlier during (curative) treatment. Oncologists fear taking away hope in patients and their families if they involve the palliative care team or talk about the worst case scenario. Parents and children on the other hand often state that they want to be informed more and sooner about this possibility, and of the opportunities pediatric palliative care can offer when curation is no longer a realistic option. 

The Netherlands has made some serious progress in the development and quality of pediatric palliative care in the last 15 years. From no formal structure for pediatric palliative care 15 years ago, to seven pediatric palliative care teams, regional networks of collaborating professionals from different disciplines and organisations from hospitals to homecare, and a Dutch Knowledge Center for pediatric palliative care, which includes a physician’s support center for dilemma’s regarding end of life. Furthermore, a format for an individual care plan was established, and the first national evidence-based guideline for pediatric palliative care was developed. The latter is now being updated and expanded, and will be implemented in 2022. At the same time, much effort was put in to developing a formal education in pediatric palliative care for nurses, doctors and other health care professionals working with children in need of palliative care. The article by Vallianatos et al. [1] nicely demonstrates what a country can do if all stakeholders join hands to improve the quality of pediatric palliative care. 

Pediatric palliative care begins with the patient and his/her family, and their quality of life, needs, worries and hopes. Zhukovsky et al. looked at the symptom and illness experience of children that are being treated for advanced cancer, and interviewed the children as well as the parents [2]. They report the impact that symptoms and cancer treatment has on their daily lives and relationships. Notably, similar themes emerged in the English- and Spanish-speaking children and parents. 

The study of Mekelenkamp et al. nicely demonstrates the aforementioned difficulty of getting the pediatric palliative care team involved earlier in the disease trajectory [3]. They first looked at the place and cause of death of transplanted hematopoietic stem cell transplantation (HSCT) patients, and secondly performed a survey in which they assessed the availability of and views on specialized pediatric palliative care services (SPPCS) among 98 HSCT professionals from 54 centers in 23 countries. Although more than 90% of health care professionals indicated that HSCT patients should have access to SPPCS, less than half of the physicians routinely referred children to a SPPCS. 

Most pediatric palliative care teams provide care for children with different underlying diseases, malignant as well as non-malignant. Baumann et al. looked at the symptoms of 89 terminally ill children, nearly half of whom had an oncological disease [4]. They found that oncological patients had a significantly higher symptom burden at the end of life, the intensity of which increased as underlying disease progressed, and the likelihood of experiencing pain and nausea/vomiting was significantly higher in the oncological patients. This is something worth considering when developing a pediatric palliative care team in a setting with limited resources, as a health care professional with ample experience in symptom control in oncology patients might be an advantage. 

Pediatric palliative care is a gratifying discipline which needs specialized health care professionals. Improvements are underway, sometimes fast, sometimes slow. However, we have a long way to go. Let us keep in mind the famous British words: “Keep calm and carry on”. 
